# Nitrogenase Fe Protein: A Multi-Tasking Player in Substrate Reduction and Metallocluster Assembly

**DOI:** 10.3390/molecules27196743

**Published:** 2022-10-10

**Authors:** Markus W. Ribbe, Kamil Górecki, Mario Grosch, Joseph B. Solomon, Robert Quechol, Yiling A. Liu, Chi Chung Lee, Yilin Hu

**Affiliations:** 1 Department of Molecular Biology and Biochemistry, University of California, Irvine, CA 92697-3900, USA; 2 Department of Chemistry, University of California, Irvine, CA 92697-2025, USA

**Keywords:** nitrogenase, Fe protein, reductase, catalysis, biosynthesis, FeS enzyme, C_1_ substrate reduction

## Abstract

The Fe protein of nitrogenase plays multiple roles in substrate reduction and metallocluster assembly. Best known for its function to transfer electrons to its catalytic partner during nitrogenase catalysis, the Fe protein is also a key player in the biosynthesis of the complex metalloclusters of nitrogenase. In addition, it can function as a reductase on its own and affect the ambient reduction of CO_2_ or CO to hydrocarbons. This review will provide an overview of the properties and functions of the Fe protein, highlighting the relevance of this unique FeS enzyme to areas related to the catalysis, biosynthesis, and applications of the fascinating nitrogenase system.

## 1. Introduction

Nitrogen is an essential element for all living organisms, being a constituent of amino acids, nucleic acids, and other indispensable organic molecules. Constituting 78% of the atmosphere, the dinitrogen (N_2_) gas is by far the most abundant source of nitrogen on Earth; however, N_2_ is not directly accessible to most living organisms and must undergo transformation into a reduced, bioaccessible form, ammonia (NH_3_). Industrially, such a transformation is enabled by the Haber–Bosch process, which is achieved at high temperatures and pressures due to the energetically demanding reaction to break the N≡N triple bond. In nature, however, the reduction of N_2_ to NH_3_ can be accomplished by a select group of microbes called diazotrophs under ambient conditions. Designated the biological nitrogen fixation, this process is facilitated by nitrogenase [1,2,3,4], a metalloenzyme expressed by diazotrophic microbes, in a reaction typically depicted as follows:

N_2_ + 8 e^−^ + 16 MgATP + 8 H^+^ → 2 NH_3_ + H_2_ + 16 MgADP + 16 P_i_

where ATP and ADP refer to adenosine tri- and diphosphate, respectively; and P_i_ refers to the inorganic phosphate.

**Figure 1:**
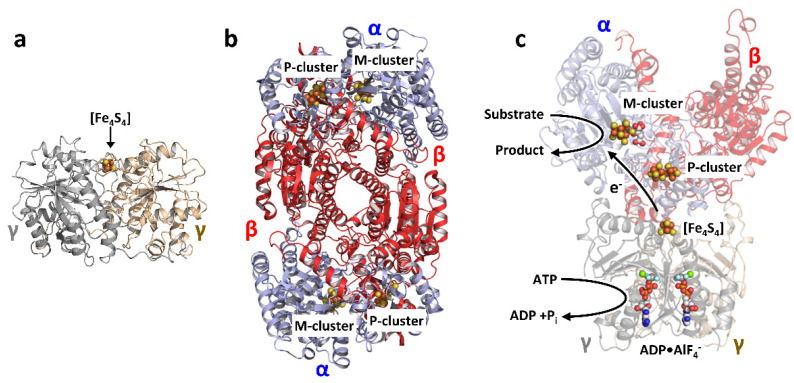
The Mo-nitrogenase of *Azotobacter vinelandii*. Crystal structures of (**a**) the nucleotide-free, γ_2_-dimeric Fe protein (PDB entry 2nip); (**b**) the α_2_β_2_-tetrameric MoFe protein (PDB entry 3u7q); and (**c**) one half of the MgADP•AlF_4_^−^-stabilized Mo-nitrogenase complex comprising one γ_2_-dimeric Fe protein and one αβ-dimer of the tetrameric MoFe protein (PDB entry 1n2c), which permits ATP-dependent, interprotein electron transfer from the [Fe_4_S_4_] cluster of the Fe protein, via the P-cluster ([Fe_8_S_7_]), to the M-cluster ([(*R*-homocitrate)MoFe_7_S_9_C]) of the MoFe protein during catalysis. The two component proteins of the Mo-nitrogenase are shown as ribbons, with the two subunits of the Fe protein colored light yellow and light gray, respectively (**a**), and the α- and β-subunits of the MoFe protein colored blue and red, respectively (**b**). The [Fe_4_S_4_] cluster of the Fe protein (**a**,**c**), the P- and M-clusters of the MoFe protein (**b**,**c**), and the MgADP•AlF_4_^−^ entity (**c**) are shown in ball-and-stick presentation, with the atoms colored as follows: Fe, orange; S, yellow; Mo, cyan; C, light gray; Mg, green; O, red; Al, dark gray; F, light blue; P, dark orange.

Three homologous nitrogenases, designated the Mo-, V-, and Fe-only nitrogenases [5,6], have been identified to date, which is mainly distinguished by the presence or absence of a heterometal at their respective cofactor sites. Encoded by *nif* genes, the “conventional” Mo-nitrogenase from *Azotobacter vinelandii* is the best-characterized member of this enzyme family, which consists of two components. One of them, designated the Fe protein, is a γ_2_-homodimer of ~60 kDa that contains a subunit-bridging [Fe_4_S_4_] cluster and a MgATP-binding site per subunit (Figure 1a) [7,8]. The other, designated the MoFe protein, is an α_2_β_2_ heterotetramer of ~230 kDa that contains a pair of complex metalloclusters within each αβ-dimer: a P-cluster ([Fe_8_S_7_]) that is situated at the α/β-subunit interface, and an M-cluster (or cofactor; [(*R*-homocitrate)MoFe_7_S_9_C]) that is located within the α-subunit (Figure 1b) [9,10]. During catalysis, the two-component proteins form a functional complex, wherein electrons are transferred concomitantly with ATP hydrolysis from the [Fe_4_S_4_] cluster of the Fe protein, via the P-cluster, to the M-cluster of the MoFe protein, where substrate reduction takes place (Figure 1c) [1,2,3,4,11]. The “alternative” V- and Fe-only nitrogenases, like their Mo-counterpart, use two-component systems to effect substrate reduction. Encoded by *vnf* and *anf* genes, respectively, the component proteins of the V- and Fe-only nitrogenases share a good degree of sequence and structural homology with their respective counterparts in the Mo-nitrogenase; in particular, the Fe protein components of the three nitrogenases, encoded by *nifH*, *vnfH* and *anfH*, respectively, are highly similar in structure and function despite displaying distinct features on their own [5,6].

**Figure 2:**
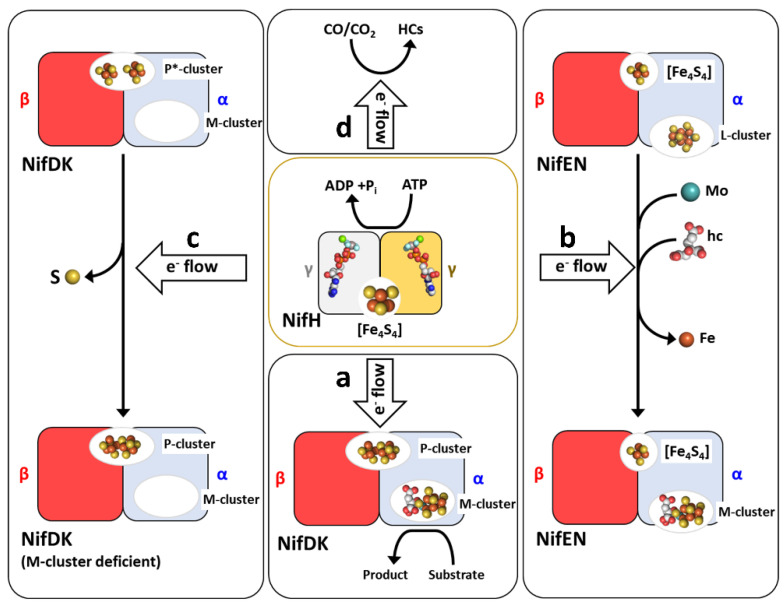
The many roles of the Fe protein (NifH) in substrate reduction and metallocluster assembly. Shown are the functions of NifH as (**a**) an electron donor for its catalytic partner, MoFe protein (NifDK), in nitrogenase catalysis; (**b**) a Mo/homocitrate (hc) insertase for the maturation of an L-cluster ([Fe_8_S_9_C]) into a fully assembled M-cluster ([(*R*-homocitrate)MoFe_7_S_9_C]) on a biosynthetic scaffold, NifEN; (**c**) a reductase for the reductive coupling of a P*-cluster (an [Fe_4_S_4_]-like cluster pair) into a P-cluster ([Fe_8_S_7_]); and (**d**) an independent reductase for the reduction of CO and/or CO_2_ into hydrocarbons (HCs). All functions of Fe protein require an electron source (**a**–**d**), with its functions in nitrogenase catalysis (**a**) and biosynthesis (**b**,**c**) also rely on ATP hydrolysis. In comparison, the function of Fe protein as a reductase on its own requires an electron source, but it can be accomplished with or without MgATP (**d**). For the purpose of simplicity, only one αβ-dimer is shown for the tetrameric NifDK (**a**), its cofactor-deficient variant (**c**), and NifEN (**b**), with each αβ-dimer interacting with one NifH dimer. The subunits, clusters, and MgADP•AlF_4_^−^ are presented and colored as those in Figure 1.

Historically, much of the attention has been focused on the catalytic component of nitrogenase because of its remarkable feat to catalyze some of the most challenging chemical transformations at its active-site cofactor under ambient conditions. However, studies in the last decade have revealed that other than functioning as the obligate electron donor for the MoFe protein during nitrogenase catalysis (Figure 2a), the Fe protein of the Mo-nitrogenase also plays a key role in the assembly of both complex metalloclusters of its catalytic partner, serving as a Mo/homocitrate insertase for the maturation of the M-cluster (Figure 2b) [12,13,14] as well as an ATP-dependent reductase for the maturation of the P-cluster (Figure 2c) [14,15,16,17,18]. Moreover, the homologous Fe proteins of both bacterial and archaeal origins were shown to act as reductases on their own and catalyze the reduction of CO_2_ and/or CO to hydrocarbons (Figure 2d) [19,20,21,22,23,24] These findings not only establish the Fe protein as a crucial multi-tasking player in nitrogenase catalysis and assembly but also point to the utility of this protein as a simple FeS template for the future development of biotechnological applications that recycle CO_2_ (a greenhouse gas) and CO (a carbon waste) into the useful hydrocarbon products.

This review will provide a brief overview of the structural and redox properties of the Fe protein, followed by a detailed discussion of the multiple roles of this unique FeS enzyme in substrate reduction and metallocluster biosynthesis. 

## 2. Structural and Redox Properties of the Fe Protein

### 2.1. The Fe Protein of the “Conventional” Mo-Nitrogenase

#### 2.1.1. Structural Features 

**Figure 3:**
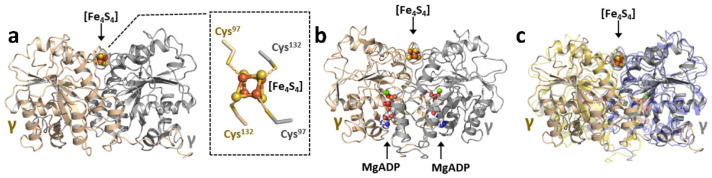
The structural impact of MgADP binding on NifH. Crystal structures of (**a**) nucleotide-free (PDB entry 2nip) and (**b**) MgADP-bound forms of NifH (PDB entry 6n4l); and (**c**) an overlay of the structures of nucleotide-free and MgADP-bound NifH, showing little change in the overall conformation of NifH upon nucleotide binding. The ligands coordinating the [Fe_4_S_4_] cluster of NifH are shown as sticks in the inset (**a**). The subunits, clusters, and MgADP are presented and colored as those in Figure 1.

The first crystal structure was reported for the *nifH*-encoded Fe protein (designated NifH) from *A. vinelandii* in 1992 [7] and, since then, this protein has been crystallized in several oxidation states, with or without nucleotide, in complex with its catalytic partner, or with its substrate CO_2_ [8,11,20,21,25,26,27]. The crystal structure of the nucleotide-free, dithionite-reduced NifH (Figure 3a) reveals the location of an [Fe_4_S_4_] cluster on a two-fold rotation axis at the subunit interface, with the cluster coordinated by two cysteines (Cys97 and Cys132) from each subunit. The cluster is nearly surface exposed, a feature that facilitates its function both as an electron donor to its catalytic NifDK partner and as a reductase on its own to enable substrate reduction (see below). Other than the cluster-binding site, there is a Walker A motif between residues 9–16 of NifH, which signifies a nucleotide binding site within each subunit. Interestingly, the crystal structures of NifH with and without MgADP [8] overlay nearly completely with each other (Figure 3b), indicating minor conformational changes of this protein upon nucleotide binding. However, spectroscopic and biochemical studies point to the conformational flexibility of this protein upon nucleotide binding in the solution that often escapes observation in the crystalline state. 

**Figure 4:**
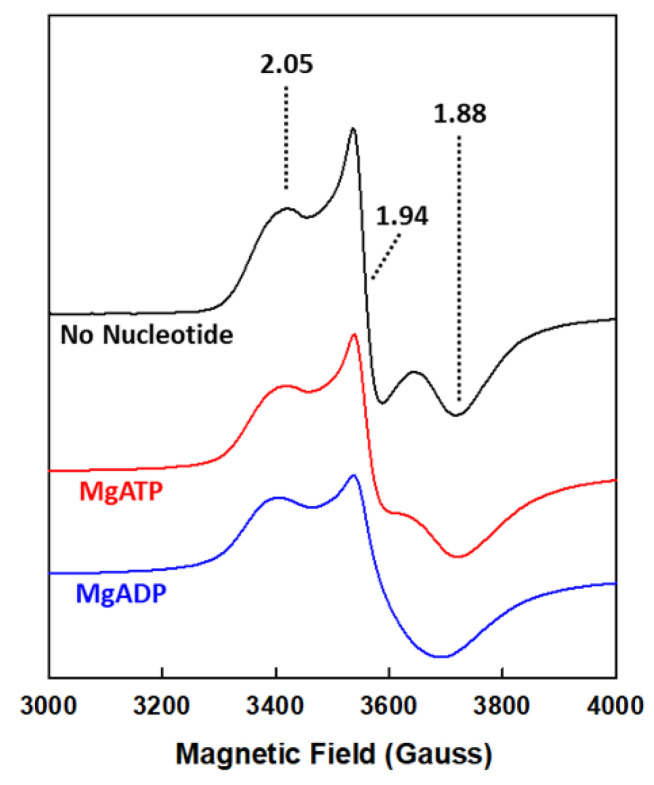
The nucleotide-free and bound states of NifH. Shown are the perpendicular-mode EPR spectra of the nucleotide-free (black), MgADP-bound (red), and MgATP-bound (blue) forms of NifH in the dithionite-reduced, [Fe_4_S_4_]^1+^ state. The *g* values are indicated.

Consistent with a conformational change upon nucleotide binding, there is a change in the line shape of the EPR signals of the MgADP- and MgATP-bound forms of NifH (Figure 4) [1,28], indicating long-distance signal transduction upon binding of the nucleotide at a location within the protein that is ~20 Å away from the [Fe_4_S_4_] cluster near the protein surface. The spectroscopic changes of NifH are accompanied by a decrease in the reduction potential of its [Fe_4_S_4_] cluster by ~100 mV upon binding of either MgADP or MgATP [29]. Chelation assays further demonstrate a limited or lack of ability for chelating agents, such as bathophenanthrolinedisulfonate or 2,2-bipyridine, to chelate the Fe atoms from the [Fe_4_S_4_] cluster of NifH in the absence of MgATP, but a full capacity of the same chelating agents to rapidly remove the Fe atoms from the NifH-associated [Fe_4_S_4_] cluster in the presence of MgATP [30,31,32]. These observations suggest that binding of MgATP renders the [Fe_4_S_4_] cluster of NifH more surface exposed and, consequently, better positioned for the inter-protein electron transfer between NifH and NifDK within the nitrogenase complex, which is in agreement with the general function proposed for the interaction between MgATP and NifH during catalysis. 

Importantly, there is a difference in the behaviors of NifH upon binding of MgADP and MgATP. Chelation experiments indicate that the [Fe_4_S_4_] cluster of NifH remains protected from chelating agents in the presence of MgADP [33,34], contrary to the substantially increased accessibility of the NifH-associated cluster to chelating agents in the presence of MgATP. SAXS analysis further reveals a substantial conformational rearrangement of NifH upon binding of MgATP; in contrast, there is a lack of conformational change upon binding of MgADP [35]. These findings align well with previous results that indicate a milder structural impact of binding of MgADP than that of MgATP on NifH [1] as well as a tighter binding of MgADP than MgATP to NifH [1] that is consistent with the fact that MgADP—the product of MgATP hydrolysis during catalysis—also serves as an inhibitor to regulate the activity of nitrogenase. Taken together, these results indicate a nucleotide-induced conformational change of NifH that is particularly relevant to the function of NifH as an ATP-dependent electron donor to NifDK within the nitrogenase complex (see Section 3.1 below). Interestingly, NifH is believed to interact analogously with an NifDK variant involved in P-cluster assembly, and with a biosynthetic scaffold that is structurally homologous to NifDK to facilitate M-cluster maturation. Both of these events are ATP-dependent, pointing to the relevance of the nucleotide-induced conformational change of NifH to its functions in nitrogenase biosynthesis (see Section 3.2 below).

#### 2.1.2. Redox Properties 

**Figure 5:**
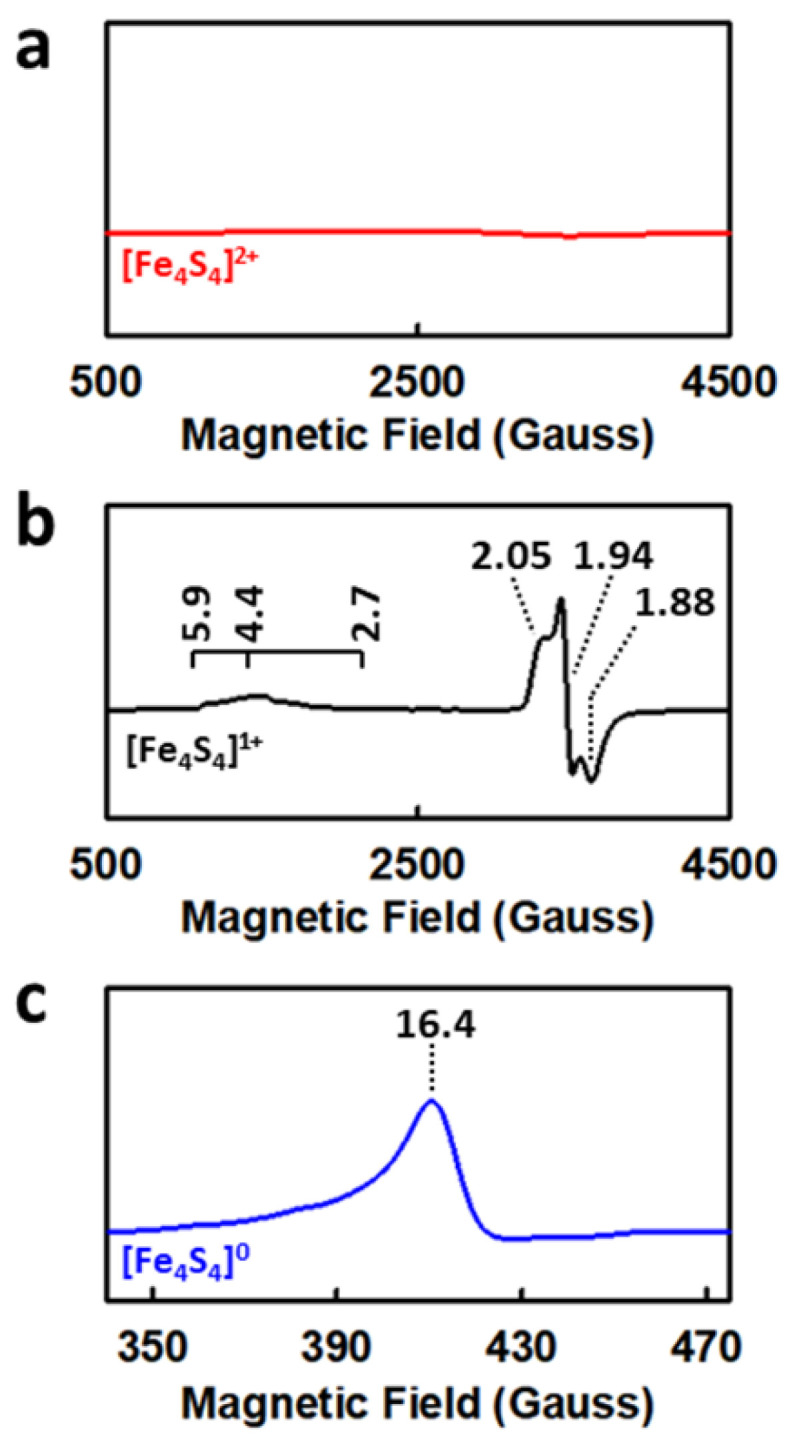
The three oxidation states of NifH. Shown are the perpendicular-mode EPR spectra of (**a**) the oxidized, [Fe_4_S_4_]^2+^ state and (**b**) the reduced, [Fe_4_S_4_]^1+^ state of NifH; and (**c**) the parallel-mode EPR spectrum of the super-reduced, all-ferrous [Fe_4_S_4_]^0^ state of NifH. The *g* values are indicated.

NifH can adopt three oxidation states: the oxidized, [Fe_4_S_4_]^2+^ state; the reduced, [Fe_4_S_4_]^1+^ state; and the super-reduced, all-ferrous [Fe_4_S_4_]^0^ state. Generated in vitro by an oxidant (e.g., IDS) and a reductant (e.g., dithionite), respectively, the oxidized [Fe_4_S_4_]^2+^ cluster is a diamagnetic species with an *S* = 0 spin state [36], whereas the reduced [Fe_4_S_4_]^1+^ cluster is a mixture of *S* = 3/2 and *S* = 1/2 species (Figure 5a,b) that can undergo interconversion upon treatment with chemical additives such as glycerol or urea [36]. Under in vivo conditions, the [Fe_4_S_4_] cluster of NifH is believed to cycle between the [Fe_4_S_4_]^2+^ and [Fe_4_S_4_]^1+^ states to enable a one-electron transfer event during nitrogenase catalysis, using flavodoxins or ferredoxins as the physiological electron donors to access the [Fe_4_S_4_]^1+^ state [37,38,39]. 

Such an interconversion between the [Fe_4_S_4_]^2+^ and [Fe_4_S_4_]^1+^ states of NifH, depicted as the “Fe protein cycle”, is one key component of the mechanistic model of nitrogenase that focuses on the interprotein electron transfer from NifH to NifDK (see Section 3.1 below). However, the question as to whether NifH can facilitate a two-electron transfer event during catalysis was raised upon the discovery of the super-reduced, all-ferrous [Fe_4_S_4_]^0^ state of NifH [40,41,42,43]. Accessed under in vitro conditions upon treatment of NifH with a strong reductant such as Ti^III^-citrate [40] or Eu^II^-DTPA [20,28], the [Fe_4_S_4_]^0^ cluster displays a characteristic, parallel-mode EPR signal at *g* = 16.4 (Figure 5c) [40] that is associated with a distinctive pink hue [43]. Assigned as an *S* = 4 ground state spin state by combined Mössbauer, EPR, and DFT studies [44,45], this super-reduced [Fe_4_S_4_]^0^ species is quite rare in nature, with the only other known example being the 2-hydroxyglutaryl-coenzyme A dehydratase of *Acidaminococcus fermentans*, which catalyzes the reversible syn-elimination of water from (*R*)-2-hydroxyglutaryl-coenzyme A [46]. While coupling such a “doubly reduced” [Fe_4_S_4_]^0^ cluster (instead of the “singly reduced” [Fe_4_S_4_]^1+^ cluster) with the oxidized [Fe_4_S_4_]^2+^ cluster would reduce the expenditure of MgATP by half and is therefore highly beneficial from the perspective of cellular energy conservation, the low reduction potential required to generate the “pink all-ferrous state” of NifH [43] has cast doubt on whether such a state can be achieved under in vivo conditions. 

Progress has been made, however, toward establishing the potential physiological relevance of the all-ferrous state of the Fe protein. A recent study demonstrated that contrary to its native counterpart, a variant of the *A. vinelandii* NifH protein containing an [Fe_4_Se_4_] cluster in place of the [Fe_4_S_4_] cluster readily displayed the all-ferrous specific, *g* = 16.4 EPR signal in the presence of dithionite (*E_1/2_* = −0.47 at 2 mM), a solution potential where the native [Fe_4_S_4_] cluster was only capable of adopting the reduced [Fe_4_S_4_]^1+^ state [47]. This observation implies that the Fe protein could potentially access the all-ferrous state under certain physiological conditions. Consistent with this suggestion, it was reported that the *vnfH*-encoded Fe protein (VnfH) from *Methanosarcina acetivorans* displayed 50% of maximum intensity of the all-ferrous specific EPR signal at −0.59 V, a potential well within the range of the physiological reduction potentials within the cell [19]. The “pink all-ferrous state” of the Fe protein was further correlated with the ability of this protein to catalyze the reduction of C_1_ substrates (see Section 3.3 below); however, experimental evidence is yet to be acquired to address whether this all-ferrous state could be used to drive nitrogenase catalysis under in vivo conditions. In this context, it should be noted that another [Fe_4_S_4_]^0^ species—generated with an in vivo electron donor, flavodoxin—has been reported previously. Characterized by biochemical, NMR, and EPR analyses, this all-ferrous species is brown in color and proposed to adopt an *S* = 0 spin state [42]. This observation is important, as the proposed all-ferrous species was obtained with a physiologically relevant reductant. However, given the EPR-silent nature of the *S* = 0 spin state, more sensitive magnetic measurements, such as those by Mössbauer spectroscopy, are required to verify the spin state of this “brown all-ferrous state”, along with further experiments that probe its physiological relevance to nitrogenase catalysis. 

### 2.2. The Fe Proteins of the “Alternative” V- and Fe-Only Nitrogenases

**Figure 6:**
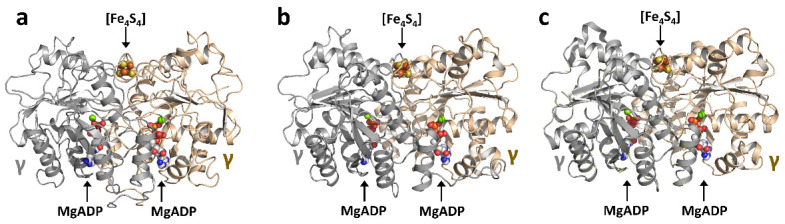
The homologous Fe proteins from Mo-, V-, and Fe-only nitrogenases. Crystal structures of the MgADP-bound forms of (**a**) NifH (PDB entry 6n4l), (**b**) VnfH (PDB entry 6q93) and (**c**) AnfH from *A. vinelandii* (PDB entry 7qqa). The subunits, clusters, and MgADP are presented and colored as those in Figure 1.

The *vnfH*- and *anf*-encoded Fe proteins (designated VnfH and AnfH) from *A. vinelandii* share a good degree of sequence homology with their *nifH*-encoded counterpart; however, the sequence identity between VnfH and NifH (~90%) is much higher than that between AnfH and NifH (~60%), pointing to a more distant relationship between AnfH and its VnfH and NifH homologs [48]. Interestingly, such a difference in the sequence is not reflected in the crystal structures of these related Fe proteins, as a structural comparison of NifH, VnfH, and AnfH in the MgADP-bound state reveals that they adopt nearly identical conformations (Figure 6) [8,49,50]. All three Fe proteins are homodimers of ~60 kDa, with their subunits having the same Rossmann-type βαβ-fold. Additionally, all three proteins have a subunit-bridging [Fe_4_S_4_] cluster that is ligated by a pair of cysteines from each subunit (Cys98 and Cys 133 in VnfH; Cys97 and Cys131 in AnfH), as well as a nucleotide-binding site within each subunit that consists of a Walker A motif [49,50]. 

Consistent with the observation of structural conservation among NifH, VnfH, and AnfH, all three Fe proteins display a mixture of *S* = 3/2 and *S* = 1/2 EPR resonances in the dithionite-reduced state [51], indicating that they contain highly similar [Fe_4_S_4_] clusters. XAS/EXAFS analysis provides further support for this argument, showing nearly indistinguishable conformations adopted by the [Fe_4_S_4_]^1+^ clusters in all three Fe proteins, except for a greater out-of-plane bend of the two NifH-associated [Fe_2_S_2_] rhomboids than those of their counterparts in VnfH and AnfH [51]. The similarity among the homologous Fe proteins extends further to their redox behaviors. Like NifH, VnfH can adopt three oxidation states, namely, the oxidized [Fe_4_S_4_]^2+^ state, the reduced [Fe_4_S_4_]^1+^ state, and the super-reduced, all-ferrous [Fe_4_S_4_]^0^ state [28]. Such an ability to adopt the same charge states are also conserved among the homologous Fe proteins of bacterial (e.g., NifH and VnfH from *A. vinelandii*) and archaeal (e.g., NifH and VnfH from *M. acetivorans*) origins [28]. In the case of AnfH, however, it is not known whether this Fe protein can adopt the same oxidation states as its NifH and VnfH counterparts, although a similar redox behavior is expected given the similar structural and electronic properties among the three homologous Fe proteins. 

As is the case with NifH, both VnfH and AnfH likely utilize the [Fe_4_S_4_]^2+/1+^ couple to facilitate a one-electron transfer event to their respective catalytic partners during nitrogenase catalysis; whereas the super-reduced [Fe_4_S_4_]^0^ state of VnfH, like that of NifH, is associated with the ability of Fe protein to perform C_1_ substrate reduction. It is interesting to note that, while VnfH displays strong cross-reactivity with NifDK—the catalytic component of Mo-nitrogenase—in substrate reduction, AnfH appears to interact poorly with NifDK in such a “hybrid” nitrogenase system, which could originate from the relatively lower sequence identity between AnfH and its VnfH and NifH homologs [48]. Moreover, when compared in the context of the complete nitrogenase, the AnfH-supported nitrogenase system is also the least active in substrate reduction, with an average activity of ~65% and 40%, respectively, relative to those of the VnfH- and NifH-supported nitrogenase systems [3]. However, comparable activities of all three Fe proteins with NifDK have also been reported [51], suggesting that further studies are required to unveil the functional similarities and distinctions among these homologous members of the Fe protein family.

## 3. The Multi-Tasking Roles of Fe Protein in Catalysis and Biosynthesis 

The Fe protein has a multitude of functions in substrate reduction and metallocluster assembly. To date, most of the studies concerning the functions of Fe protein have been conducted on NifH, leading to the recognition of its functions as (1) an obligate electron donor to its catalytic partner, NifDK, during nitrogenase catalysis (Figure 2a) [1,2,3,4]; (2) an Mo/homocitrate insertase to enable M-cluster maturation (Figure 2b) [12,13,14,15]; and (3) a reductase to facilitate P-cluster formation (Figure 2c) [15,16,17,18]. Additionally, the NifH and VnfH proteins from *A. vinelandii* and *M. acetivorans* have been characterized for their functions as independent reductases to effect the ambient conversion of C_1_ substrates, including CO_2_ and CO, to hydrocarbons (Figure 2d) [19,20,21,22,23,24]. The multi-tasking functions of the Fe protein will be discussed in detail below.

### 3.1. The Role of Fe Protein in Nitrogenase Catalysis

**Figure 7:**
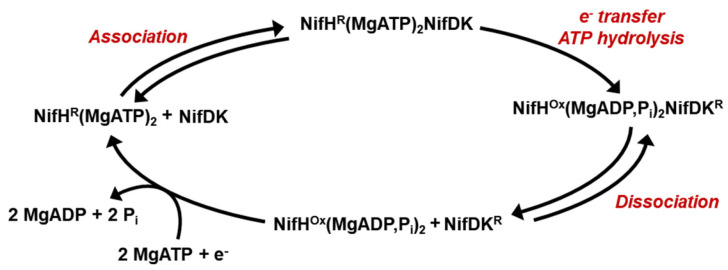
The Fe protein cycle. This cycle depicts the function of NifH as a reductase component of nitrogenase to facilitate the interprotein electron transfer, concomitant with ATP hydrolysis, to its catalytic partner NifDK via repeated association and dissociation. NifH^R^, reduced NifH; NifH^Ox^, oxidized NifH; NifDK^R^, reduced NifDK.

NifH is best known for its role as an obligate electron donor to its catalytic partner, NifDK, during nitrogenase catalysis. The catalytic behavior of NifH is depicted by a so-called “Fe protein cycle” (Figure 7) [1], which begins with the binding of the reduced, MgATP-bound NifH to NifDK, which facilitates the inter-protein electron transfer from NifH to NifDK concomitant with the hydrolysis of MgATP. Subsequently, the oxidized, MgADP/P*_i_*-bound NifH is dissociated from the reduced NifDK, and MgADP and P*_i_* are released from NifH. This event is followed by a re-reduction of NifH and a “reloading” of MgATP on this protein, which initiates the next cycle of complex formation and electron transfer between NifH and NifDK. The “Fe protein cycle” must repeat multiple times before substrate reduction by NifDK can take place through a so-called “MoFe protein cycle” [1,3,52,53], which involves a stepwise intra-protein delivery of protons and electrons to the M-cluster of NifDK for the binding, activation, and reduction of substrates.

**Figure 8:**
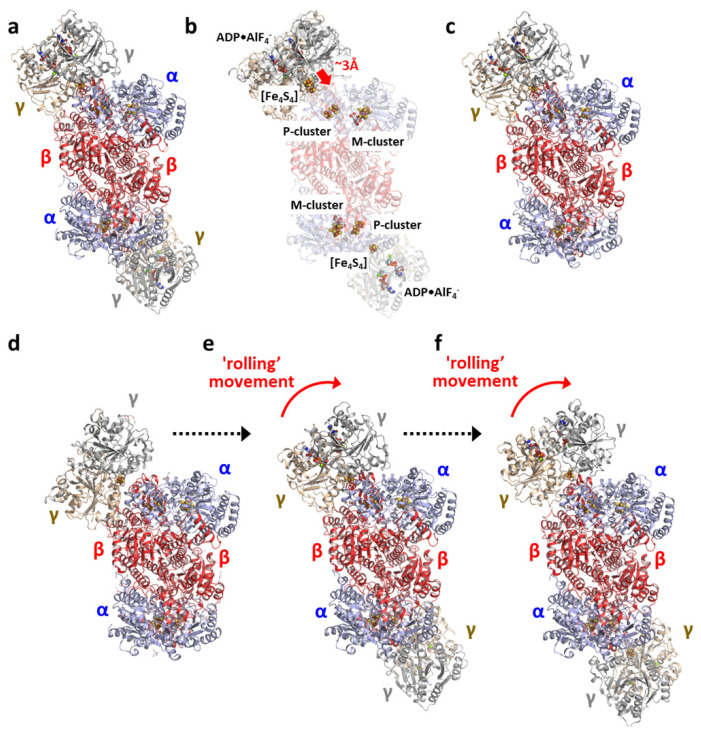
The various Mo-nitrogenase complexes from *A. vinelandii*. (**a**,**b**) Crystal structures of the MgADP•AlF_4_^−^ stabilized complex (**a**) alone (PDB entry 1n2c), and (**b**) overlaid with the nucleotide-free NifH (PDB entry 2nip), showing a movement of the [Fe_4_S_4_] cluster of NifH by ~3 Å (indicated by a red arrow) toward the P-cluster of NifDK upon complex formation. (**c**) Cryo-EM structure of turnover (TO) complex I, showing a molar ratio of 1:1 between NifH and NifDK (PDB entry 7ut8). (**d**–**f**) Crystal structures of complexes generated (**d**) without nucleotide (PDB entry 2afh); (**e**) with MgADP (PDB entry 2afi); and (**f**) with MgAMPPCP (PDB entry 2afk), showing a plausible “rolling” movement of NifH across the surface of NifDK upon hydrolysis of MgATP. Note that the molar ratio between NifH and NifDK is 2:1 within the complexes generated with non-hydrolyzable MgATP analogs, MgADP•AlF_4_^−^ (**a**) and MgAMPPCP (**f**), and with MgADP (**e**), contrary to the molar ratio of 1:1 between NifH and NifDK within the complexes generated with MgATP under turnover conditions (**c**), and without nucleotide (**d**). The subunits, clusters, MgADP•AlF_4_^−^, MgADP and MgAMPPCP are presented and colored as those in Figure 1.

The interaction between NifH and NifDK was initially explored by docking analysis and cross-linking between NifH and NifDK [54,55] which led to the identification of the Glu112 residue of NifH as a potential docking site. Subsequent mutagenic analyses revealed the potential involvement of several surface residues of NifH, including Arg100, Arg140, and Lys143, in the interprotein transfer of electrons from NifH to NifDK [56,57,58,59]. These earlier findings were validated in 1997 by the crystal structure of a “transition state” complex of *A. vinelandii* Mo-nitrogenase, which was stabilized with MgADP·AlF_4_^−^, a non-hydrolyzable MgATP analog (Figure 8a) [11]. Interestingly, while the overall conformation of NifH in this complex is similar to that of the non-complexed NifH, there is a clear movement of its [Fe_4_S_4_] cluster toward the protein surface (Figure 8b), which places this cluster more closely to the P-cluster in NifDK. Subsequent crystallographic attempts led to the observation of a number of complexes between NifH and NifDK, with NifH present in the nucleotide-free, MgADP-bound, and MgAMPPCP (another non-hydrolyzable MgATP analog)-bound states (Figure 8d–f) [27]. A comparison of these complexes reveals a “rolling” movement of NifH across the surface of NifDK that presumably occurs concomitantly with MgATP hydrolysis and electron transfer (Figure 8d–f). 

Notably, the Mo-nitrogenase complexes crystallized with MgADP and non-hydrolyzable MgATP analogs (i.e., MgADP·AlF_4_^−^ and MgAMPPCP) all consist of NifH and NifDK at a molar ratio of 2:1. In contrast, a recent cryo-EM study of the *A. vinenaldii* Mo-nitrogenase complex, which was generated with MgATP under turnover conditions, revealed a molar ratio of 1:1 between NifH and NifDK (Figure 8c) [60] Consistent with this finding, crystallographic analyses of an N_2_-bound NifDK from *A. vinelandii* demonstrated an asymmetric binding of N_2_ to the two cofactors, leading to the hypothesis of an alternating docking of NifH on the two αβ-dimers of NifDK that results in an asynchronous sequence of events of N_2_ reduction in the two cofactors [61]. While further evidence is required to substantiate this hypothesis, these recent structural observations illustrate a highly dynamic interaction between NifH and NifDK during substrate turnover that underlies the intricate substrate reduction mechanism of nitrogenase.

### 3.2. The Role of Fe Protein in Nitrogenase Biosynthesis

The role of NifH in nitrogenase biosynthesis was first recognized from the observation that the deletion of *nifH* resulted in an NifDK species that was not only M-cluster-deficient but also contained a precursor in place of a mature P-cluster [16]. Combined biochemical, spectroscopic, and structural analyses have established NifH as a Mo/homocitrate insertase for M-cluster maturation (Section 3.2.1) [12,13,14,15], and as a reductase for P-cluster formation (Section 3.2.2) [15,16,17,18]. Strikingly, the actions of NifH in nitrogenase catalysis (see above) and metallocluster biosynthesis (see below) are analogous to each other in that both involve electron transfer in an ATP-dependent manner. However, there are clear distinctions between the functionality of NifH in catalysis and assembly. Apart from the distinctive nature of substrate reduction and cluster assembly that underlies the differences between these processes, a recent study has demonstrated the requirement of a more negative reduction potential for NifH to fulfill its function in nitrogenase catalysis than those for the same protein to carry out its functions in nitrogenase assembly [47]. Moreover, while the formation of the P-cluster involves interactions between NifH and an NifDK variant that are analogous to those between NifH and NifDK during catalysis, maturation of the M-cluster occurs on a biosynthetic scaffold that is homologous to, but clearly distinct from NifDK, and involves more chemical transformations than those occurring during the process of P-cluster maturation. The distinct functions of NifH in these biosynthetic processes will be discussed in detail below. 

#### 3.2.1. M-cluster Maturation

**Figure 9:**
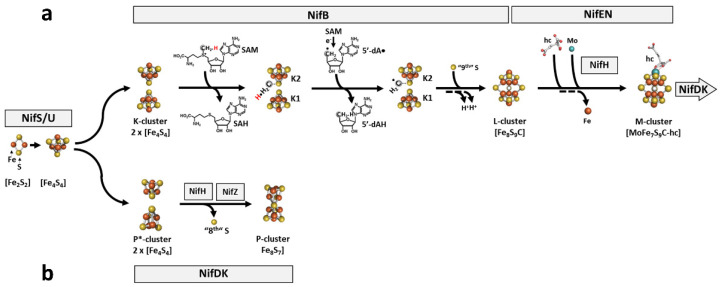
Biosynthesis of the complex metalloclusters of Mo-nitrogenase. Shown are (**a**) the “*ex situ*” assembly of the M-cluster, where NifH serves as a Mo/homocitrate (hc) insertase to mature the L-cluster ([Fe_8_S_9_C]) into a fully assembled M-cluster ([(*R*-homocitrate)MoFe_7_S_9_C]) on NifEN; and (**b**) the “*in situ*” assembly of the P-cluster, where NifH serves as a reductase to facilitate the reductive coupling of a P*-cluster (an [Fe_4_S_4_]-like cluster pair) into a fully assembled P-cluster ([Fe_8_S_7_]) on NifDK. Note that the assembly pathways of M- and P-clusters share the early steps that involve the sequential formation of [Fe_2_S_2_] and [Fe_4_S_4_] clusters by the concerted actions of NifS and NifU. The two pathways branch after these early events, with a pair of [Fe_4_S_4_] clusters (K-cluster), delivered to NifB for the formation of an M-cluster, and a pair of [Fe_4_S_4_] clusters (P*-cluster) delivered to NifDK for the formation of a P-cluster. The biosynthetic events on NifB involve radical SAM-dependent coupling of the two 4Fe units of the K-cluster (designated K1 and K2) into an 8Fe L-cluster concomitant with the insertion of a “9th sulfur” (**a**); whereas the biosynthetic events on NifDK involve the stepwise formation of the two P-clusters in the two αβ-halves of NifDK via reduction coupling of the two 4Fe units of the P*-cluster into an [Fe_8_S_7_] P-cluster concomitant with the removal of an “8^th^ sulfur” (**b**).

Biosynthesis of the M-cluster (Figure 9a) occurs outside its destined binding site in NifDK (“*ex situ*”) and involves a series of *nif*-encoded assembly scaffolds [14,15]. This process begins with the actions of NifS and NifU, with NifS serving as a pyridoxal phosphate-dependent cysteine desulfurase for the formation of a protein-bound cysteine persulfide that is subsequently donated to NifU for the sequential formation of [Fe_2_S_2_] and [Fe_4_S_4_] clusters [62,63,64,65]. Subsequently, a pair of [Fe_4_S_4_] clusters (designated the K-cluster) is transferred to a radical SAM enzyme, NifB, and undergoes radical SAM-dependent transformation into an [Fe_8_S_9_C] precursor (designated the L-cluster) that is indistinguishable in structure to the fully assembled M-cluster except for the absence of Mo and homocitrate (Figure 9a) [66,67,68,69,70,71,72,73]. Subsequently, the L-cluster is transferred to the next biosynthetic scaffold, NifEN, where it is matured into an M-cluster upon insertion of Mo and homocitrate (Figure 9a) [12,13,74,75,76,77,78,79,80,81,82] prior to the transfer of the matured M-cluster to its target location within NifDK (Figure 9a) [83]. 

Although the involvement of NifH in M-cluster maturation was implicated by the observation that deletion of *nifH* resulted in a cofactor-deficient from NifDK [16], the specific function of NifH in this process had remained elusive until an NifEN protein was purified from an *nifHDK*-deletion strain of *A. vinelandii* and shown to be bound with an [Fe_8_S_9_S] precursor to the M-cluster (i.e., the L-cluster) [81,82]. Subsequent biochemical and EPR studies demonstrated that the L-cluster on NifEN could be converted to a fully complemented M-cluster upon incubation with NifH, MgATP, molybdate, homocitrate, and dithionite [12,13,79]. Fe and Mo K-edge XAS analyses provided further validation that the NifEN-associated M-cluster was structurally nearly identical to its NifDK-associated counterpart except for an asymmetric ligation of Mo that likely originated from a different protein environment in NifEN than that in NifDK [13]. Re-isolation of NifH following incubation with molybdate, homocitrate, MgATP, and dithionite revealed that this protein was “loaded” with Mo and homocitrate and that this “loaded” form of NifH could be directly used as a Mo/homocitrate source to convert the L-cluster to an M-cluster on NifEN [12]. Together, these results have firmly established NifH as an ATP-dependent Mo/homocitrate insertase that plays a key role in M-cluster maturation.

The requirement of MgATP and reductant for NifH to carry out its function in M-cluster maturation points to a certain analogy between the functions of NifH in cofactor assembly and substrate catalysis. Such an analogy is not surprising given the homology in the overall structure and cluster composition of NifEN and NifDK. An α_2_β_2_ heterotetramer, NifEN houses an [Fe_4_S_4_] cluster in place of the P-cluster ([Fe_8_S_7_]) at the α/β-subunit interface, and an L-cluster ([Fe_8_S_9_C]) in place of an M-cluster ([(*R*-homocitrate)MoFe_7_S_9_C]) within the α-subunit [78]. It is likely, therefore, that the MgATP-dependent interaction between NifH and NifEN during M-cluster maturation resembles that between NifH and NifDK during substrate reduction. Adding to the similarity between the biosynthetic complex (NifH/NifEN) and the catalytic complex (NifH/NifDK) is the function of NifH as an ATP-dependent reductase during these processes. Only in the case of M-cluster maturation, the reducing equivalent is, at least in part, used for the reduction of molybdate, as the Mo K-edge XAS spectrum of the “loaded” NifH revealed a decreased number of Mo = O bonds (2–3 instead of 4 in molybdate) and reduction in the effective nuclear charge of Mo due to a change in the formal oxidation state or the ligation of Mo [12]. Along this line of observation, a nucleotide-dependent reduction of orthovanadate by NifH was also reported [84], pointing to a general role of this type of reaction in facilitating the activation and reduction of transition metals. It is also possible that additional reduction events occur through the nucleotide-dependent, interprotein transfer of electrons between NifH and NifEN to facilitate M-cluster maturation on NifEN, much like that between NifH and NifDK during catalysis. However, further experiments are required to test this hypothesis.

Consistent with the binding of the metal alongside the organic compound, the EPR spectrum of Mo- and homocitrate-“loaded” NifH adopts a line shape between those of the MgADP- and MgATP-bound states of NifH [12]. This observation coincides with that derived from the first crystal structure of an ADP-bound form of NifH, wherein Mo occupies a position that corresponds to the γ-phosphate of ATP [7]. Given the structural analogy between phosphate and molybdate, such an ADP-Mo binding mode could represent the initial attachment of Mo to NifH, a concept supported by similar nucleotide-assisted processes proposed for the insertion of Mo into the pterin-based cofactors [85]. The delivery of Mo and homocitrate appears to be concerted, as maturation of the L-cluster on NifEN can only occur when Mo and homocitrate are supplied simultaneously. The reason behind this effect, however, requires further investigation. 

#### 3.2.2. P-cluster Formation

Biosynthesis of the P-cluster (Figure 9b) [16,17,18,86,87,88,89,90,91,92], like that of the M-cluster, begins with the sequential formation of [Fe_2_S_2_] and [Fe_4_S_4_] units by NifS and NifU [15]. However, further maturation of small FeS units into a P-cluster takes place at its target location within NifDK (“*in situ*”), with NifH playing a key role in this process [15,93]. 

Earlier attempts to extract P-clusters from NifDK have resulted in the recovery of [Fe_4_S_4_] clusters, pointing to the possibility that the P-cluster is generated from [Fe_4_S_4_] modules. Consistent with this suggestion, biochemical, EPR, and XAS studies of an NifDK variant isolated from an *nifH*-deletion strain of *A. vinelandii* revealed that this variant (designated Δ*nifH* NifDK) was not only cofactor-deficient but also contained a pair of [Fe_4_S_4_]-like clusters in place of the [Fe_8_S_7_] P-cluster [16,17]. VTVH-MCD analysis demonstrated that each [Fe_4_S_4_] pair in Δ*nifH* NifDK comprised an [Fe_4_S_4_]^1+^ cluster and a diamagnetic [Fe_4_S_4_]-like cluster, which became paramagnetic upon oxidation by IDS [94]. Biochemical experiments further established the [Fe_4_S_4_]-like cluster pair (designated the P*-cluster) as a physiologically relevant precursor to the P-cluster, as Δ*nifH* NifDK could be fully activated upon incubation with NifH, MgATP, dithionite, and solvent-extracted M-clusters [18]. Interestingly, monitoring of the real-time P-cluster maturation by Fe K-edge XAS experiments led to the observation of a stepwise formation of the two P-clusters in Δ*nifH* NifDK; in other words, the two P-clusters are matured, one at a time, in the two αβ-halves of Δ*nifH* NifDK [90]. Interestingly, such a sequential assembly of the two P-clusters in NifDK was also captured in three cofactor-deficient NifDK variants generated upon strategic deletions of genes encoding biosynthetic proteins, with (*i*) Δ*nifH* NifDK (containing two P*-clusters) representing the starting conformation; (*ii*) Δ*nifB*Δ*nifZ* NifDK (containing one P*-cluster and one P-cluster) representing the intermediary conformation; and (*iii*) Δ*nifB* NifDK (containing two P-clusters) representing the end conformation of the stepwise assembly of P-clusters in NifDK [15]. Importantly, the asymmetry in P-cluster assembly parallels that proposed for substrate reduction on the basis of the recent observation of asymmetric N_2_-bound conformations in the two cofactors of NifDK [61], which is not particularly surprising as both processes likely involve an alternating docking of NifH on the two αβ-halves of its respective NifDK partner. 

The strict dependence of P-cluster maturation on the concentration of reductant and hydrolysis of ATP [90] points to a crucial role of NifH in supplying electrons for the reductive coupling of two 4Fe subclusters (P*-cluster) into an 8Fe cluster (P-cluster). This argument was supported by Fe K-edge XAS experiments that demonstrated a successive reduction in the Fe atoms of the P*-cluster during the maturation process until they eventually stabilized at a near all-ferrous oxidation state in the matured P-cluster [90]. SAXS analyses further revealed a 6 Å gap at the α/β-dimeric interface of ∆*nifH* NifDK (containing the P*-cluster) that was not observed in ∆*nifB* NifDK (containing the P-cluster), suggesting a role of NifH in holding the α- and β-subunits of NifDK together while donating electrons for the reductive coupling of two 4Fe units into an 8Fe entity [92]. A dual role of NifH in facilitating electron transfer while providing structural aid for P-cluster maturation was also supported by the observation of a sole requirement of NifH for the assembly of the “first” P-cluster, but a requirement of both NifH and NifZ for the assembly of the “second” P-cluster in Δ*nifB*Δ*nifZ* NifDK [86,90,91,95]. This observation was rationalized by the “second” [Fe_4_S_4_]-like cluster pair being forced apart upon fusion of the “first” pair of [Fe_4_S_4_]-like clusters and, consequently, a need to have NifH stabilizing the “second” α/β-subunit interface—along with NifZ in a chaperone-like function—while facilitating electron transfer for the coupling of the “second” [Fe_4_S_4_] cluster pair into an [Fe_8_S_7_] P-cluster. However, the exact function of the small, metal-free NifZ in this process remains elusive.

### 3.3. The Role of Fe Protein in C_1_ Substrate Reduction 

**Figure 10:**
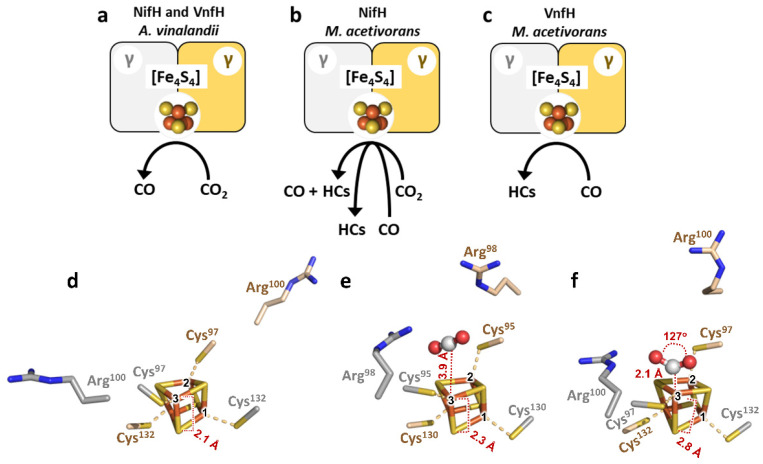
Differential reactivities of the Fe proteins with C_1_ substrates. (**a**–**c**) Schematic presentations showing the abilities of (**a**) the *A. vinelandii* NifH and VnfH proteins to reduce CO_2_ to CO; (**b**) the *M. acetivorans* NifH protein to reduce CO_2_ to CO and hydrocarbons (HCs), and CO to hydrocarbons (HCs); and (**c**) the *M. acetivorans* VnfH protein to reduce CO to hydrocarbons (HCs). (**d**–**f**) Crystal structures of (**d**) the reduced *Av*NifH (PDB entry 2nip) that is free of substrate and (**e**) the reduced *Ma*NifH with CO_2_ bound in an unactivated, linear conformation (PDB entry 6nzj); and (**f**) DFT-optimized structure of the super-reduced *Av*NifH (PDB entry 6o0b) with CO_2_ bound in an activated, bent conformation.

The ability of the Fe protein to reduce C_1_ substrates was initially observed for the NifH and VnfH proteins from *A. vinelandii* under both in vitro and in vivo conditions. Both *A. vinelandii* Fe proteins can reduce CO_2_ to CO (Figure 10a) [24], with or without ATP, in the presence of dithionite; however, the yields of CO are higher in both cases in the presence of ATP, likely due to a decrease in the reduction potentials of their associated [Fe_4_S_4_] clusters by ~100 mV upon ATP binding [24]. Substitution of dithionite (*E*^0^ = −0.47 V at 2 mM) with Eu^II^-DTPA (*E*^0^ = −1.14 V vs. SHE at pH 8) in the in vitro assays led to a dramatic increase in the yields of CO by both proteins, and repeated addition of Eu^II^-DTPA resulted in a total turnover number of 8 for both NifH and VnfH [24]. A substantial increase in activity could also be accomplished by both NifH and VnfH in the whole cells of *A. vinelandii*, a trait attributed to the reducing intracellular environment that protects the Fe protein from O_2_ damage, and the assortment of ferredoxins and/or flavodoxins that serve as the “correct” electron donors to the Fe protein to effect in vivo reduction of CO_2_ [24]. EPR analysis revealed a decrease in the intensities of the characteristic signals of the [Fe_4_S_4_]^1+^ and [Fe_4_S_4_]^0^ clusters, respectively, in the reactions driven by dithionite and Eu^II^-DTPA, consistent with oxidation of both clusters to an EPR-silent [Fe_4_S_4_]^2+^ state upon the donation of electrons to CO_2_. Additionally, such a change in the oxidation state of the [Fe_4_S_4_] cluster was accompanied by the appearance of a new EPR feature at *g* = 1.99 in both dithionite- and Eu^II^-DTPA-driven reactions, which likely resulted from an interaction between the [Fe_4_S_4_] cluster and the substrate, CO_2_ [24]. These observations collectively point to the redox-active [Fe_4_S_4_] cluster of Fe protein as the active-site cofactor for CO_2_ reduction. 

Interestingly, the two Fe proteins from *A. vinelandii* cannot reduce CO_2_ past CO to hydrocarbons; instead, they are capable of oxidizing CO to CO_2_ at a much higher efficiency than CO_2_ reduction, making them functionally resemblant to the Ni-containing CO dehydrogenase [96,97,98]. In comparison, the NifH and VnfH proteins from *M. acetivorans* cannot oxidize CO to CO_2_; yet, both proteins can reduce C_1_ substrates to hydrocarbons (Figure 10b,c) despite a difference in their reactivities with CO or CO_2_ [23]. Specifically, *Ma*NifH can reduce CO_2_ to CO and C_1_–C_3_ hydrocarbons, and CO to C_1_–C_4_ hydrocarbons, in the presence of Eu^II^-DTPA; whereas *Ma*VnfH is incapable of reducing CO_2_ but can reduce CO to C_1_-C_4_ hydrocarbons with a product yield that is one magnitude lower than that of *Ma*NifH under the same reaction conditions. The differential C_1_ substrate reactivities of *Ma*NifH and *Ma*VnfH have been shown to be associated with the strengths (i.e., reduction potentials) of their respective all-ferrous states in C_1_ substrate reduction, with *Ma*VnfH having a weaker all-ferrous state at a more positive reduction potential than the stronger all-ferrous state adopted by its *Ma*NifH counterpart at a more negative reduction potential [19]. Consistent with this observation, a variant of *Av*NifH containing an [Fe_4_Se_4_] cluster in place of the [Fe_4_S_4_] cluster was shown to adopt the all-ferrous state at a more positive potential than its native *Av*NifH counterpart and, consequently, reduce CO_2_ to CO at a reduced efficiency than the latter in the presence of Eu^II^-DTPA, where both Fe proteins existed fully in the super reduced, all ferrous state [47]. 

The mechanism of the Fe protein-facilitated reduction of CO_2_ to CO was probed by structural, mutagenic, and theoretical studies of *Av*NifH and *Ma*NifH (Figure 10d–f) [20,21], leading to the proposal of an asymmetric utilization of the seemingly symmetrical elements of the Fe protein and its associated [Fe_4_S_4_] cluster for the binding, activation, and reduction of CO_2_. The proposed pathway of CO_2_ reduction begins with the formation of a cage-like configuration by a conserved Arg pair at the protein surface, which traps the unactivated, linear CO_2_ molecule near the [Fe_4_S_4_] cluster at a distance of ~4 Å between the C atom of CO_2_ and one of the Fe atoms (designated Fe3) of the [Fe_4_S_4_] cluster (Figure 10e). Subsequently, the CO_2_ moiety is activated into a bent, carboxylate-like conformation by an asymmetric movement of one Arg residue (designated proximal Arg) toward one O atom of CO_2_, which allows hydrogen-bonding interactions between them, as well as an asymmetric coordination of the C atom of CO_2_ by the unique Fe3 atom of the cluster (Figure 10f). Reduction of CO_2_ then proceeds asymmetrically, with the proximal Arg serving as the proton donor that facilitates proton-coupled electron transfer to CO_2_, leading to the scission of a C-O bond concomitant with the removal of O as water, followed by the release of CO as the product of the reaction [20,21]. 

The mechanism of the FeS-based reduction of CO_2_ to hydrocarbons was further examined by DFT calculations of the reaction catalyzed by the synthetic [Fe_4_S_4_] cluster in the presence of a strong reductant, SmI_2_ (*E*^0′^ = −1.55 V vs. SCE in tetrahydrofuran) [23]. One energetically plausible pathway derived from these calculations begins with binding of CO_2_ to the all-ferrous [Fe_4_S_4_]^0^ cluster (*S* = 0), followed by protonation and proton-coupled electron transfer to the CO_2_ moiety that results in the scission of one C-O bond concomitant with the removal of O as water, similar to that proposed for the protein-facilitated reduction of CO_2_ to CO. Subsequently, CO is either released or transformed into a cluster-bound, Fe-formyl intermediate, which undergoes further reduction to yield a reactive Fe-methyl species. Such a species is either reduced and released as CH_4_ or proceeds to C-C coupling via migratory insertion of CO into the Fe-methyl bond. The resulting Fe-acetyl intermediate then undergoes a series of reduction steps to yield an Fe-ethyl intermediate, followed by further reduction of this intermediate and release of C_2_H_6_ as the product of the first C-C coupling reaction. The proposal of an aldehyde-like, Fe-formyl intermediate was validated by the experimentally observed reduction of formaldehyde (CH_2_O) to CH_4_ by the free [Fe_4_S_4_] cluster in solutions [23]; whereas the proposal of C-C coupling via migratory insertion of CO into a metal-alkyl bond is consistent with that proposed for the chemical Fischer–Tropsch process, highlighting a broad similarity between the FeS-catalyzed Fischer–Tropsch-like reaction and its chemical counterpart while pointing to the possibility of developing enzymatic FeS catalysts for the ambient recycling of the greenhouse gas (CO_2_) and carbon waste (CO) into hydrocarbons in the future.

## 4. Conclusions

The Fe protein is a versatile FeS enzyme that plays multiple roles in substrate reduction and metallocluster assembly. Other than serving as the canonical redox partner to NifDK during catalysis, NifH also plays a key role in the assembly of both M- and P-clusters of NifDK, interacting with its respective biosynthetic partners in a manner analogous to, yet distinct from its interaction with NifDK during substrate reduction. Importantly, all these processes have a common denominator in ATP hydrolysis and electron transfer, the quintessential function of NifH as an ATP-dependent reductase. Moreover, the recent discovery that the Fe protein can effect the ambient reduction of CO_2_ and CO to hydrocarbons adds another function to the catalytic repertoire of this unique FeS protein, making it a potential template for the future development of FeS catalysts for the carbon-neutral production of hydrocarbon products. The many functions of the Fe protein await further exploration to provide answers related to the catalysis, biosynthesis, and applications of the fascinating nitrogenase enzyme.
